# Multi-Fading Factor and Updated Monitoring Strategy Adaptive Kalman Filter-Based Variational Bayesian

**DOI:** 10.3390/s21010198

**Published:** 2020-12-30

**Authors:** Chenghao Shan, Weidong Zhou, Yefeng Yang, Zihao Jiang

**Affiliations:** 1 College of Intelligent Systems Science and Engineering, Harbin Engineering University, Harbin 150001, China; shanchenghao123@hrbeu.edu.cn (C.S.); jiangzihao@hrbeu.edu.cn (Z.J.); 2 Center for Control Theory and Guidance Technology, Harbin Institute of Technology, Harbin 150001, China; 18B904013@stu.hit.edu.cn

**Keywords:** variational Bayesian, multiple-fading factors, time-varying noise covariance matrices, inaccurate noise, target tracking, updated monitoring strategy

## Abstract

Aiming at the problem that the performance of adaptive Kalman filter estimation will be affected when the statistical characteristics of the process and measurement of the noise matrices are inaccurate and time-varying in the linear Gaussian state-space model, an algorithm of multi-fading factor and an updated monitoring strategy adaptive Kalman filter-based variational Bayesian is proposed. Inverse Wishart distribution is selected as the measurement noise model and the system state vector and measurement noise covariance matrix are estimated with the variational Bayesian method. The process noise covariance matrix is estimated by the maximum a posteriori principle, and the updated monitoring strategy with adjustment factors is used to maintain the positive semi-definite of the updated matrix. The above optimal estimation results are introduced as time-varying parameters into the multiple fading factors to improve the estimation accuracy of the one-step state predicted covariance matrix. The application of the proposed algorithm in target tracking is simulated. The results show that compared with the current filters, the proposed filtering algorithm has better accuracy and convergence performance, and realizes the simultaneous estimation of inaccurate time-varying process and measurement noise covariance matrices.

## 1. Introduction

In many practical engineering applications, the actual values of the required state variables are often not directly available. For example, when radar detects an air target, it can calculate the target distance based on information such as reflected waves. Still, there is random interference in the radar detection process, resulting in random noise in the observation signal. In this case, it is impossible to obtain the required state variables accurately, and these state variables can only be estimated or predicted based on the observed signal. In linear systems, the Kalman filter is the optimal filter [1]. With the development of computer technology, the calculation requirements and complexity of Kalman filtering no longer become obstacles to its application [2]. At present, the Kalman filtering theory has been widely used in tracking, navigation, guidance, and other areas [3,4,5,6,7,8,9].

The application of the Kalman filter requires prior knowledge of the mathematical model of the system and the statistical characteristics of noise. Still, in many practical application problems, these are unknown or only partially known [10,11,12]. If inaccurate mathematical models or statistical noise characteristics are used to design the Kalman filter, the performance of the filter will be degraded, resulting in larger estimation errors, and even filter divergence. To solve this problem, various adaptive Kalman filters (AKF) have been produced [13,14].

The Sage–Husa filter (SH-KF) is widely used because of its simple algorithm, which can estimate the first and second moments of noise online [15]. However, the Sage–Husa adaptive noise estimator has problems such as a large amount of calculation and easy divergence of state estimation [16]. In addition, some literature has pointed out that the covariance matrices of process noise and observation noise cannot be estimated dynamically in real-time by the Sage–Husa adaptive estimator at the same time, which can only estimate the other noise covariance matrix when one noise covariance matrix is known [17,18]. The maximum-likelihood-based adaptive filtering method (ML-KF) can evaluate and correct the second-order moments of the noise statistics online, but it needs to rely on accurate innovation covariance estimation, and ML-KF requires a large sliding window to obtain a precise estimate of the noise covariance matrix, which theoretically makes it only for time-varying noise covariance matrix estimation [19,20]. Strong tracking Kalman filter (ST-KF) is an adaptive filtering algorithm based on the principle of residual orthogonality. It adjusts the weight of new measurement data by adding an estimate of the one-step predictive covariance matrix. It has strong robustness regarding model parameter mismatch, lower sensitivity to the statistical characteristics of noise and initial values, and a strong ability to track sudden changes. But its adjustment ability for each filtering channel is the same, and state predicted error covariance matrix (PECM) and measurement noise covariance matrix (MNCM) are not estimated [21,22]

In recent years, many scholars have introduced the variational Bayesian machine-learning method into the KF algorithm and proposed AKF algorithm based on the variational Bayesian approach (VB-KF), which is an approximation of the Bayesian method. By choosing a suitable conjugate prior distribution, the slow time-varying measurement noise covariance can be estimated [23,24,25]. Literature [26] proposed a variational adaptive Kalman filter (R-VBKF) but only estimated the system state vector and the measurement noise covariance matrix (MNCM); the accuracy is not satisfactory enough. In the algorithm presented in the literature [27], the state predicted error covariance matrix (PECM) and the measurement noise covariance matrix (MNCM) are estimated, but the process noise covariance matrix (PNCM) is not directly assessed. The specific estimated value of the PNCM cannot be obtained, and the estimation accuracy needs to be further improved.

Aiming at the linear Gaussian state–space model with slow time-varying covariance of process and measurement noise, taking into account the estimation accuracy, convergence performance, robustness, and the realization of simultaneous estimation of noise covariance matrices, the multi-fading factor and updated monitoring strategy, AKF-based variational Bayesian (MFMS-VBAKF) is proposed. Its feasibility is proved by simulation experiments.

The main contributions of the algorithm proposed in this paper are as follows: compared with the inverse-Gamma distribution applied by the R-VBKF algorithm in [25], the algorithm proposed in this paper selects the distribution of the measurement noise model as a more reasonable inverse-Wishart distribution in variational Bayesian approach, which improves the estimation accuracy of system state vector and MNCM; compared with SH-KF in [15], the algorithm proposed in this paper guarantees the positive semi-definiteness of the PNCM under the maximum posterior estimation by designing an updated monitoring strategy, and achieves the simultaneous estimation of the PNCM and MNCM.; and compared with ST-KF [21], the proposed algorithm has different adjustment abilities for each channel by introducing multi-fading factors and the PECM estimation accuracy is also improved.

The main structure is as follows: Section 2 illustrates the mathematical modeling of the problem. In Section 3, the multi-fading factor and updated monitoring strategy AKF-based variational Bayesian is derived. In Section 4, the proposed algorithm is compared with the existing algorithm through the simulation of the target tracking application, and the excellent performance of the proposed algorithm is proved. Section 5 summarizes the conclusions. Finally, Section 6 plans the future work.

## 2. Problem Modeling

Consider the following discrete linear stochastic system of the state–space model

(1) $$\begin{array}{c} X_{k}=\Phi_{k-1}X_{k-1}+\omega_{k-1}, \end{array}$$

(2) $$\begin{array}{c} Z_{k}=H_{k-1}X_{k}+v_{k}, \end{array}$$

where (1) and (2) are process and measurement equations, respectively. k is discrete-time, $X_{k}\in R^{n\times n}$ is the state vector of the system at time k, $Z_{k}\in R^{m\times m}$ is the measurement signal vector of the corresponding state. $\Phi_{k}\in R^{n\times n}$ is the state-transition matrix, $H_{k}\in R^{m\times n}$ is the measurement matrix. $\omega_{k}\in R^{n}\;$and $v_{k}\in R^{m}$ are uncorrelated white Gaussian noise with zero mean vectors and covariance matrices $Q_{k}$ and $R_{k},$ respectively. The initial state $X_{0}$ is assumed to be a Gaussian distribution with mean vector ${\hat{X}}_{0}$ and the covariance matrix $P_{0}$. $X_{0}$ is uncorrelated to $\omega_{k}$ and $v_{k}$ at any time [1].

For linear Gaussian state–space models, the Kalman filter (KF) algorithm is an optimal estimation filter algorithm. If the noise covariance matrices $Q_{k}$ and $R_{k}$ are fully known, KF estimates the state vector $X_{k}$ through the measurement information of $Z_{1:k}$, and the estimation accuracy is satisfactory. However, the performance of the KF algorithm overly depends on the prior knowledge of the noise statistics. If the time-varying noise covariance matrices $Q_{k}$ and $R_{k}\;$are unknown or inaccurate, the accuracy of the KF algorithm will decrease, and even cause the estimation to diverge. Besides, when most existing AKF algorithms estimate the PNCM $Q_{k}$ and MNCM $R_{k}$ at the same time, the filtering will diverge. Therefore, a multi-fading factor and updated monitoring strategy AKF-based variational Bayesian with inaccurate time-varying PNCM and MNCM is proposed.

## 3. The Proposed Multi-Fading Factor and Updates Monitoring Strategy AKF-Based Variational Bayesian

In the VBAKF algorithm, the independent state vector $X_{k}$ and the measurement noise covariance matrix $R_{k}$ are regarded as the parameters to be estimated.

### 3.1. AKF-Based Variational Bayesian (VBAKF)

#### 3.1.1. Prediction Process and Distribution Selection

In the traditional Kalman filter framework, the Gaussian distributions are selected as the distributions of one-step predicted probability density function (PDF) $P(X_{k}|Z_{1:k-1})$ and likelihood PDF $p(Z_{k}|X_{k})$:

(3) $$\begin{array}{c} p\left(X_{k}|Z_{1:k-1}\right)=N\left(X_{k-1};{\hat{X}}_{k:k-1},P_{k:k-1}\right), \end{array}$$

(4) $$\begin{array}{c} p\left(Z_{k}|X_{k}\right)=N\left(Z_{k};H{\hat{X}}_{k:k-1},R_{k}\right), \end{array}$$

where N(G;μ,Σ) is the Gaussian distribution and μ and Σ represent the mean and variance of the distribution, respectively. The PDF of the Gaussian distribution is:

(5) $$\begin{array}{c} N\left(G;\mu ,\Sigma \right)=\frac{1}{\sqrt{\left|2\pi \Sigma \right|}}exp^{-\frac{1}{2}{\left(G-\mu \right)}^{Τ}\Sigma^{-1}\left(G-\mu \right)}, \end{array}$$

According to Equation (1), the predicted state vector ${\hat{X}}_{k:k-1}$ and the corresponding one-step predicted error covariance matrix (PECM) $P_{k:k-1}$ can be described as:

(6) $$\begin{array}{c} {\hat{X}}_{k:k-1}=\Phi_{k-1}{\hat{X}}_{k-1:k-1}, \end{array}$$

(7) $$\begin{array}{c} P_{k:k-1}=\Phi_{k-1}P_{k-1:k-1}\Phi_{k-1}{}^{T}+Q_{k-1}, \end{array}$$

where ${\hat{X}}_{k:k-1}$ and $P_{k:k-1}$ represent the state estimation at time k−1 and the corresponding estimation error covariance matrix, respectively. ${\left(.\right)}^{T}$ represents the transpose of the matrix. Among them, it is assumed that the real PNCM $Q_{k}$ is unknown due to the complex environmental factors in real applications, which will lead to an inaccurate $P_{k:k-1}$ in Equation (7). The estimation methods for $Q_{k}$ and $P_{k:k-1}$ will be given in the next two sections.

In this section, the aim is to infer $X_{k}$ with $R_{k}$. For the purpose, the conjugate prior distributions need to be first selected for inaccurate MNCM $R_{k}$ since the conjugacy can ensure that the posterior distribution and the prior distribution maintain the same functional form.

According to Bayesian statistical theory, if the Gaussian distribution has a known mean, the conjugate prior distribution of the covariance matrix can be regarded as the inverse Wishart (IW) distribution [28]. $C^{-1}$ is the inverse matrix of a positive definite matrix C. If $C^{-1}$ follows the Wishart-distribution $W\left(C^{-1};\lambda ,\Psi^{-1}\right)$, the matrix C follows the IW distribution:

(8) $$\begin{array}{c} IW\left(C;\lambda ,\Psi \right)=\frac{{\left|\Psi \right|}^{\lambda /2}}{2^{\lambda d/2}\Gamma_{d}\left(\frac{\lambda }{2}\right)}{\left|C\right|}^{-\left(\lambda +d+1\right)/2}exp^{-\frac{1}{2}tr\left[\Psi C^{-1}\right]}. \end{array}$$

In Equation (8), C is a symmetric positive definite random matrix, distribution parameter λ is a dof parameter, Ψ is a symmetric positive definite matrix, d is the dimension of C, $\Gamma_{n}\left(.\right)$ represents a multivariate gamma function, and tr[.] is the matrix trace calculation. Additionally, if λ>d+1 and $E\left[C^{-1}\right]\sim IW\left(C;\lambda ,\Psi \right)$, then $E\left[C^{-1}\right]=\left(\lambda -d-1\right)\Psi^{-1}$. E(.) stands for mathematical expectation calculation [29]. Since $R_{k}$ is the covariance matrix of the Gaussian PDF, the prior distribution of $P\left(R_{k}|Z_{1:k-1}\right)$ can be written as IW distribution:

(9) $$\begin{array}{c} p\left(R_{k}|Z_{1:k-1}\right)=IW\left(R_{k:k-1};{\hat{t}}_{k:k-1},{\hat{T}}_{k:k-1}\right), \end{array}$$

where ${\hat{t}}_{k:k-1}$ and ${\hat{T}}_{k:k-1}$ denote the degrees of freedom (dof) parameter and scale matrix of $p\left(R_{k}|Z_{k-1}\right)$, respectively. Next, the values of ${\hat{t}}_{k:k-1}$ and ${\hat{T}}_{k:k-1}$ need to be assigned.

Owing to the Bayesian theorem, the prior distribution $p\left(R_{k}|Z_{k-1}\right)$ can be written as:

(10) $$\begin{array}{c} p\left(R_{k}|Z_{1:k-1}\right)=\int^{\;}p\left(R_{k}|R_{k-1}\right)p\left(R_{k-1}|Z_{1:k-1}\right)dR_{k-1}, \end{array}$$

where $p\left(R_{k-1}|Z_{1:k-1}\right)$) is the posterior probability density function (PDF) of the MNCM $R_{k-1}$.

Utilizing (9), the distribution of posterior PDF $p\left(R_{k-1}|Z_{1:k-2}\right)$ can be replaced as inverse Wishart distribution, due to the prior distribution $p\left(R_{k-1}|Z_{1:k-1}\right)$ of MNCM $R_{k-1}$ is selected as inverse Wishart distribution, and $p\left(R_{k-1}|Z_{1:k-1}\right)$ can be written as:

(11) $$p\left(R_{k-1}|Z_{1:k-1}\right)=IW(R_{k-1};{\hat{t}}_{k-1:k-1},{\hat{T}}_{k-1:k-1}).$$

To guarantee $p\left(R_{k}|Z_{1:k-1}\right)$ also obeys the inverse Wishart distribution, a changing factor ρ is introduced to modify the one-step predicted values of the distribution parameters ${\hat{t}}_{k:k-1}$ and ${\hat{T}}_{k:k-1}$. The formulas are as follows:

(12) $$\begin{array}{c} {\hat{t}}_{k:k-1}=\rho \left({\hat{t}}_{k-1}-m-1\right)+m+1, \end{array}$$

(13) $$\begin{array}{c} {\hat{T}}_{k:k-1}=\rho {\hat{T}}_{k-1}, \end{array}$$

among them, *m* is the dimension of $Z_{k}$, ρ∈(0,1], the time-varying measurement noise covariance matrix can be changed with a certain probability distribution, and control the posterior and prior probability density functions to have the same distribution.

In addition, the initial PDF distribution of MNCM $R_{0}$ is also assumed as inverse Wishart distribution.$\;p\left(R_{0}\right)=IW\left(R_{0};{\hat{t}}_{0:0},{\hat{T}}_{0:0}\right)$. At the initial moment, to formulate the prior information of the measurement noise covariance matrix, the mean value of $R_{0}$ is considered as the initial fixed measurement noise covariance matrix ${\tilde{R}}_{0}$, i.e.,

(14) $$\begin{array}{c} {\tilde{R}}_{0}=\frac{{\hat{T}}_{k:k-1}}{{\hat{t}}_{k:k-1}-m-1} \end{array}$$

Assuming that the prior distribution of the joint probability density function of the state variable and the MNCM is the product of the Gaussian distribution and the inverse Wishart distribution, the prediction process can be defined as:

(15) $$\begin{array}{c} p\left(X,R_{k}|Z_{1:k-1}\right)=p\left(X_{k}|Z_{1:k-1}\right)p\left(R_{k}|Z_{k-1}\right)=N\left(X_{k-1};{\hat{X}}_{k:k-1},P_{k:k-1}\right)IW\left(R_{k-1};{\hat{t}}_{k-1:k-1},{\hat{T}}_{k-1:k-1}\right) \end{array}$$

#### 3.1.2. Variational Update Process

Aiming at estimating the state $X_{k}$ and the MNCM $R_{k}$, their joint posterior PDF $p\left(X_{k},R_{k}|Z_{1:k}\right)$ needs to be calculated. However, the analytical solution of this joint posterior PDF cannot be obtained directly. The variational Bayesian method is utilized to find an approximate PDF of a free form as follows [30]:

(16) $$\begin{array}{c} p(X_{k},R_{k}|Z_{1:k})\approx q\left(X_{k}\right)q\left(R_{k}\right), \end{array}$$

where q(.) means the approximate posterior PDF of p(.).

In the standard VB method, Kullback–Leibler divergence (KLD) is used to measure the degree of approximation between the approximation posterior PDF and the true posterior PDF, and the optimal solution is obtained by minimizing KLD. The VB method can provide a closed form solution for the approximate posterior PDF. Minimizing the KLD between the approximation posterior PDF$\;q\left(X_{k}\right)$, $q\left(R_{k}\right)$ and the true joint posterior$\;p\left(X_{k},R_{k}|Z_{1:k-1}\right)$ is used to form the VB-approximation [30]:

(17) $$\begin{array}{c} \left\{q\left(X_{k}\right)q\left(R_{k}\right)\right\}=\arg min\;KLD\left(q\left(X_{k}\right),q\left(R_{k}\right)\;||\;p\left(X_{k},R_{k}|Z_{1:k}\right)\right), \end{array}$$

The divergence function KLD(.) is defined as:

(18) $$\begin{array}{c} KLD\left(q\left(A\right)\;||\;p\left(A\right)\right)=\int^{\;}q\left(A\right)\log\frac{q\left(A\right)}{p\left(A\right)}dA. \end{array}$$

Combined with Equations (17) and (18), the optimal solution of Equation (16) is derived as:

(19) $$\begin{array}{c} \log q\left(X_{k}\right)=E_{R_{k}}\left[\log p\left(X_{k},R_{k},Z_{1:k}\right)\right]+c_{X_{k}}, \end{array}$$

(20) $$\begin{array}{c} \log q\left(R_{k}\right)=E_{X_{k}}\left[\log p\left(X_{k},R_{k},Z_{1:k}\right)\right]+c_{R_{k}}, \end{array}$$

where log(.) stands for natural logarithm calculation, $E_{\;\varphi }\left[.\right]$ denotes the expectation calculation of the approximate posterior PDF of the variable φ, $c_{X_{k}}$ and $c_{R_{k}}$ represent the constants of variable $X_{k}$ and MNCM $R_{k}$, respectively. The solutions of Equations (19) and (20) cannot be solved directly since $q\left(X_{k}\right)$ and $q\left(R_{k}\right)$ are coupled. Therefore, the fixed-point iteration method is introduced to calculate the solution to these parameters.

The further form of Equation (20) can be derived as (See Appendix A for details):

(21) $$\begin{array}{c} \log q^{\left(i+1\right)}\left(R_{k}\right)=c_{R_{k}}-\frac{1}{2}\left(m+{\hat{t}}_{k:k-1}+2\right)\log\left|R_{k}\right|-\\ \frac{1}{2}trace\left(\left(E^{\left(i\right)}\left[\left(Z_{k}-H_{k}X_{k}\right){\left(Z_{k}-H_{k}X_{k}\right)}^{T}\right]+{\hat{T}}_{k:k-1}\right)R_{k}^{-1}\right), \end{array}$$

where: $q^{\left(i\right)}\left(.\right)$ represents the approximate probability distribution of q(.) at the i-th iteration, trace(.) is the calculation of the matrix trace, $C_{R_{k}}$ is a constant related to $R_{k}$ which independent of the distribution form, and m is the dimension of the real observation matrix. The expectation part of Equation (21) is defined as $V_{k}^{\left(i\right)}$, and expanded as:

(22) $$\begin{array}{c} V_{k}^{\left(i\right)}=E^{\left(i\right)}\left[\left(Z_{k}-H_{k}X_{k}\right){\left(Z_{k}-H_{k}X_{k}\right)}^{T}\right]\\ =\int^{\;}\left(Z_{k}-H_{k}X_{k}\right){\left(Z_{k}-H_{k}X_{k}\right)}^{T}+N\left(X_{k};{\hat{X}}_{k:k}^{i},P_{k:k}\right)dX_{k}\\ =\left(Z_{k}-H_{k}{\hat{X}}_{k}^{i}\right){\left(Z_{k}-H_{k}{\hat{X}}_{k}^{i}\right)}^{T}+H_{k}P_{k}H_{k}^{T}, \end{array}$$

it can be seen that $q^{\left(i+1\right)}\left(R_{k}\right)$ obeys a new inverse Wishart distribution form as follows,

(23) $$\begin{array}{c} q^{\left(i+1\right)}\left(R_{k}\right)=IW\left(R_{k};{\hat{t}}_{k}^{\left(i+1\right)},{\hat{T}}_{k}^{\left(i+1\right)}\right). \end{array}$$

and the distribution parameters ${\hat{t}}_{k}^{\left(i+1\right)}$ and ${\hat{T}}_{k}^{\left(i+1\right)}$ are, respectively, as follows:

(24) $$\begin{array}{c} {\hat{t}}_{k}^{\left(i+1\right)}={\hat{t}}_{k:k-1}+1, \end{array}$$

(25) $$\begin{array}{c} {\hat{T}}_{k}^{\left(i+1\right)}=V_{k}^{\left(i\right)}+{\hat{T}}_{k}^{\left(i\right)}, \end{array}$$

Similarly, the logarithmic expression of the approximate distribution of the system state $X_{k}$ is as follows:

(26) $$\begin{array}{c} \log q^{\left(i+1\right)}\left(X_{k}\right)=-\frac{1}{2}{\left(Z_{k}-H_{k}X_{k}\right)}^{T}E^{\left(i+1\right)}\left[R_{k}^{-1}\right]\left(Z_{k}-H_{k}X_{k}\right)-\\ \frac{1}{2}{\left(X_{k}-{\hat{X}}_{k:k-1}\right)}^{T}P_{k:k-1}^{-1}\left(X_{k}-{\hat{X}}_{k:k-1}\right)+c_{X_{k}}, \end{array}$$

where $E^{\left(i+1\right)}{\left[R_{k}^{-1}\right]}^{-1}$ is given by:

(27) $$\begin{array}{c} E^{\left(i+1\right)}{\left[R_{k}^{-1}\right]}^{-1}={\left({\hat{t}}_{k}^{\left(i+1\right)}-m-1\right)}^{-1}{\hat{T}}_{k}^{\left(i+1\right)}. \end{array}$$

The likelihood PDF $p\left(Z_{k}|X_{k}\right)$ in Equation (4) after updating the (i+1)-th iteration can be derived as follows:

(28) $$\begin{array}{c} p^{\left(i+1\right)}\left(Z_{k}|X_{k}\right)=N\left(Z_{k};H_{k}X_{k},{\hat{R}}_{k}^{\left(i+1\right)}\right), \end{array}$$

The corrected measurement noise covariance matrix (MNCM) ${\hat{R}}_{k}^{\left(i+1\right)}$ can be written as:

(29) $${\hat{R}}_{k}^{\left(i+1\right)}=E^{\left(i+1\right)}{\left[R_{k}^{-1}\right]}^{-1}$$

Since $q\left(X_{k}\right)$ obeys the Gaussian distribution as $q\left(X_{k}\right)=N\left(X_{k};{\hat{X}}_{k},P_{k}\right)$. Combining with the standard Kalman filter framework, the gain matrix $K_{k}^{\left(i+1\right)}$, system state ${\hat{X}}_{k}^{\left(i+1\right)}$, and state covariance $P_{k}^{\left(i+1\right)}$ in the variational measurement update are corrected as follows, respectively:

(30) $$\begin{array}{c} K_{k}^{\left(i+1\right)}=\;P_{k:k-1}H_{k}^{T}{\left(H_{k}P_{k:k-1}H_{k}^{T}+{\hat{R}}_{k}^{\left(i+1\right)}\right)}^{-1}, \end{array}$$

(31) $$\begin{array}{c} {\hat{X}}_{k}^{\left(i+1\right)}={\hat{X}}_{k:k-1}+K_{k}^{\left(i+1\right)}\left(Z_{k}-H_{k}{\hat{X}}_{k:k-1}\right), \end{array}$$

(32) $$\begin{array}{c} P_{k}^{\left(i+1\right)}=\left(I-K_{k}^{\left(i+1\right)}H_{k}\right)P_{k:k-1}{\left(I-K_{k}^{\left(i+1\right)}H_{k}\right)}^{T}+K_{k}^{\left(i+1\right)}{\hat{R}}_{k}^{\left(i+1\right)}{\left(K_{k}^{\left(i+1\right)}\right)}^{T} \end{array}$$

Analyzing the above derivation, it can be seen that the implicit solution of the variational update formula is constituted by the Equations (22), (24), (25), and (29)–(32). The expected maximum approach is used to iteratively calculate $q\left(X_{k}\right)$ and $q\left(R_{k}\right)$ to update the parameters $X_{k}$ and $R_{k}$ to be estimated continuously. When $q\left(X_{k}\right)$ and $q\left(R_{k}\right)$ are closer to $p\left(X,R_{k-1}|Z_{1:k}\right)$, the KLD value of Equation (17) is smaller, and the estimation results of the parameter to be estimated adaptively approach to the true value until the iteration of the variational update process is finished. At this time, the optimal estimation results of parameters ${\hat{X}}_{k}$ and ${\hat{R}}_{k}$ to be estimated at time k can be calculated as follows (N is the number of fixed-point iterations):

(33) $$\begin{array}{c} q\left(X_{k}\right)\approx q^{\left(N\right)}\left(X_{k}\right)=\left(X_{k};{\hat{X}}_{k;k}^{\left(N\right)},P_{k;k}^{\left(N\right)}\right)=\left(X_{k};{\hat{X}}_{k:k},P_{k:k}\right), \end{array}$$

(34) $$q\left(R_{k}\right)\approx q^{\left(N\right)}\left(R_{k}\right)=IW\left(R_{k};{\hat{t}}_{k}^{\left(N\right)},{\hat{T}}_{k}^{\left(N\right)}\right)=IW\left(R_{k};{\hat{t}}_{k|k},{\hat{T}}_{k|k}\right).$$

### 3.2. Updated Monitoring Strategy Based on Maximum a Posterior (MAP) for Estimating the PNCM $Q_{k}$

Some existing adaptive filtering methods estimate the process noise covariance matrix (PNCM) $Q_{k}$ and measurement noise covariance matrix (MNCM) $R_{k}$ at the same time, it is easy to cause the accuracy of the estimated value of the state $X_{k}$ to decrease or even diverge. This is caused by the value of $Q_{k}$ becoming negative definite matrix during the estimation process [17].

Aiming at realizing the simultaneous estimation of the PNCM $Q_{k}$, MNCM $R_{k}$ and improving the estimation accuracy of the state vector $X_{k}$. An updated monitoring strategy based on maximum a posterior (MAP) for estimating the PNCM $Q_{k}$ is proposed.

According to the state–space model as Equations (1) and (2), in paper [15], the maximum a posteriori suboptimal unbiased estimation method based on the noise statistics of measurement $\left\{Z_{1},Z_{2},Z_{3},\cdots Z_{k}\right\}$ for estimating the PNCM $Q_{k}$ is given as:

(35) $${\hat{Q}}_{k}=\frac{1}{k}*\overset{k-1}{\underset{s=0}{\sum }}[K_{s}\gamma_{s}\gamma_{s}^{T}K_{s}^{T}+P_{s:s}-\Phi_{s-1}P_{s-1:s-1}\Phi_{s-1}^{T}],$$

combining Equation (35) with the conclusion of Section 3.1, where$\;K_{s}$=$K_{s}^{\left(N\right)}$ is the optimal gain calculated by VBAKF through Nth variational iterations at time s, $P_{s:s}=P_{s}^{\left(N\right)}\;$is the state covariance calculated after Nth variational iterations at time s and $\gamma_{s}=Z_{k}-{\hat{X}}_{k:k-1}$ is the residual.

From a statistical point of view, Equation (35) is an arithmetic average, and the weight coefficient in the formula is 1/(k+1). However, when estimating the time-varying process noise covariance matrix, the role of the latest information should be highlighted, which can be achieved by multiplying each item in $\overset{k}{\underset{s=0}{\sum }}\left[.\right]$ by a different weighting coefficient. Owing to the exponential weighting method, we can assign different weights to each item in $\overset{k}{\underset{s=0}{\sum }}\left[.\right]$, and increase the weight of the latest information, thereby improving the accuracy of ${\hat{Q}}_{k}$ estimation. The exponential weighting method is introduced, and the weighting coefficient $\left\{\vartheta_{s}\right\}$ is selected to satisfy:

(36) $$\begin{array}{c} \vartheta_{s}=\vartheta_{s-1}b;\;b\in \left(0,1\right);\;\overset{k}{\underset{s=0}{\sum }}\vartheta_{s}=1. \end{array}$$

further deduced as follows:

(37) $$\begin{array}{c} \vartheta_{s}=d_{k}b^{s},\;d_{k}=\frac{\left(1-b\right)}{\left(1-b^{k+1}\right)};\;s\in \left[0,k\right], \end{array}$$

where b is the attenuation factor. In $\overset{k}{\underset{s=0}{\sum }}\left[.\right]$ of Equation (35), each item is multiplied by the weight coefficient $d_{k}$, instead of the original weight coefficient. The time-varying process noise covariance matrix (PNCM) $Q_{k}$ estimation method is obtained, and the recursive algorithm is derived as:

(38) $$\begin{array}{c} {\hat{Q}}_{k}=\left(1-d_{k}\right){\hat{Q}}_{k-1}+d_{k}\left(K_{k}^{\left(N\right)}\gamma_{k}\gamma_{k}^{T}K_{k}^{\left(N\right){}^{T}}+P_{k}^{\left(N\right)}-\Phi_{k-1}P_{k-1}^{\left(N\right)}\Phi_{k-1}^{T}\right). \end{array}$$

Equations (7), (30)–(32), and (38) constitutes the VBAKF algorithm that simultaneously estimates the PNCM $Q_{k}$, MNCM $R_{k}$, state vector $X_{k},$ and one-step predicted state error covariance matrix (PECM) $P_{k:k-1}$.

However, through simulation experiments, the estimation of the PNCM $Q_{k}$ by the above algorithm is prone to abnormality; that is, it loses positive semi-definiteness, which leads to filtering divergence.

To solve this problem, based on not losing the information of the original process noise estimation algorithm (38), an updated monitoring strategy of process noise parameters is designed. Firstly, it is judged whether $Q_{k}$ calculated by Equation (38) is a positive semi-definite matrix. Then the adjustment factor β is introduced to update the process noise estimation parameters to ensure that the corrected PNCM meets the requirements.

The right side of the Equation (38) is shifted as follows:

(39) $$\begin{array}{c} {\hat{Q}}_{k}={\hat{Q}}_{k-1}+d_{k}\left(K_{k}^{\left(N\right)}\gamma_{k}\gamma_{k}^{T}K_{k}^{\left(N\right){}^{T}}+D_{k}\right), \end{array}$$

(40) $$\begin{array}{c} D_{k}=P_{k}^{\left(N\right)}-\Phi_{k}P_{k-1}^{\left(N\right)}\Phi_{k}^{T}-{\hat{Q}}_{k-1}. \end{array}$$

Generally speaking, the selection of the initial value of the state error covariance matrix $P_{0}$ is imprecise, and the deviation from the ideal value is large in the initial stage of filtering, resulting in the absolute value of the theoretical process noise covariance matrix $D_{k}$ determined by Equation (39) being much larger than $\gamma_{k}\gamma_{k}^{T}$. It is easy to cause ${\hat{Q}}_{k}$ to lose the positive semi-definiteness, and then the filtering will diverge. Therefore, it is necessary to introduce an adjustment factor to attenuate the effect of the state error covariance matrix at the initial moment, to avoid the indefiniteness of the estimated value of the PNCM, to prevent the filter from diverging. The updated monitoring strategy of the PNCM is as follows:

(41) $${\hat{Q}}_{k}={\hat{Q}}_{k-1}+d_{k}\left(K_{k}^{\left(N\right)}\gamma_{k}\gamma_{k}^{T}K_{k}^{\left(N\right){}^{T}}+{\left(\beta_{k}\right)}^{p}\left(P_{k}^{\left(N\right)}-\Phi_{k-1}P_{k-1}^{\left(N\right)}\Phi_{k-1}^{T}-{\hat{Q}}_{k-1}\right)\right),$$

where p≥1 is a positive integer (the initial value is 1), $\beta_{k}$ is the adjustment factor, and the value of $\beta_{k}$ is related to the state error variance matrix as follows:

(42) $$\begin{array}{c} \beta_{k}=exp^{-a_{k}}, \end{array}$$

(43) $$\begin{array}{c} a_{k}=\frac{trace\left(P_{k}^{\left(N\right)}-\Phi_{k-1}P_{k-1}^{\left(N\right)}\Phi_{k-1}^{T}-{\hat{Q}}_{k-1}\right)}{trace\left(K_{k}^{\left(N\right)}\gamma_{k}\gamma_{k}^{T}K_{k}^{\left(N\right){}^{T}}\right)}. \end{array}$$

**Figure 1:**
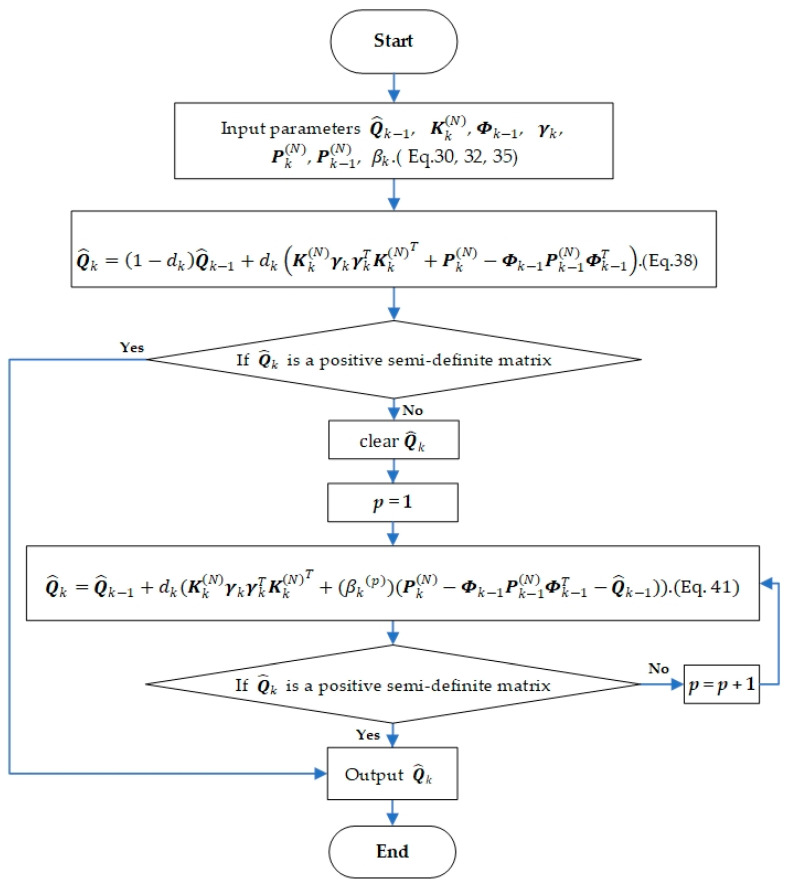
The flowchart of one time-step of the updated monitoring strategy based on maximum a posterior (MAP) for estimating the process noise covariance matrix (PNCM).

The specific process for the updated monitoring strategy is as follows: monitor the process noise covariance matrix calculated by Equation (38), and judge whether ${\hat{Q}}_{k}$ is a positive semi-definite matrix to determine whether ${\hat{Q}}_{k}$ needs to be updated. If not, output ${\hat{Q}}_{k}$. Otherwise, turn to Equation (41) and set p=1; Equation (41) is used to update the process noise parameters. Continue the monitoring of the updated estimated process noise covariance matrix to determine whether it is necessary to continue to update. If it is necessary, take p=p+1; Equation (41) is used to recalculate ${\hat{Q}}_{k}$. The loop is executed until ${\hat{Q}}_{k}$ is a positive semi-definite matrix. End the update of the process noise covariance matrix at the current moment. The flowchart of one time-step of the updated monitoring strategy is shown in Figure 1.

So far, combined with the traditional Kalman filter framework, the VBAKF algorithm with updated monitoring strategy is derived to estimate system state vector $X_{k}$, the process noise covariance matrix (PNCM) $Q_{k}$, measurement noise covariance matrix (MNCM) $R_{k}$, one-step predicted state error covariance matrix (PECM) $P_{k:k-1}$, and state error covariance matrix $P_{k}$ at the same time.

### 3.3. Improved by Introducing Multiple Fading Factors

If the statistical characteristics of the process and measurement noise are time-varying, the convergence speed of VBAKF will slow down, and there will be a particular error in the estimation result, which will be reflected by the residual sequence $\gamma_{k}$ [31].

In view of this, to improve the accuracy of estimation, the multiple fading factor $L_{md}$ is introduced to realize the correction of the one-step predicted error covariance matrix (PECM) $P_{k:k-1}$. Equation (7) can be rewritten as:

(44) $$\begin{array}{c} P_{k:k-1}^{*}=L_{md}\Phi_{k-1}P_{k-1}\Phi_{k-1}{}^{T}+{\hat{Q}}_{k-1}, \end{array}$$

adjusting the gain $K_{k}^{\left(N\right)}$ in real-time to keep $\gamma_{k}$ orthogonal, forcing the filter to keep track of the actual state of the system. The tracking ability is thereby improved [31]. The calculation method of multiple fading factors $L_{md}$ is as follows:

(45) $$\begin{array}{c} L_{md}=diag\left[\lambda_{k}^{1},\lambda_{k}^{2},\cdots ,\lambda_{k}^{n}\right], \end{array}$$

(46) $$\begin{array}{c} \lambda_{k}^{i}=\{\begin{array}{rr} \lambda_{k}^{0i}, & \lambda_{k}^{0i}>1\\ 1, & \lambda_{k}^{0i}\leq 1 \end{array}\;i=1,2,\cdots ,n, \end{array}$$

where n is the dimension of the state vector $X_{k}$, $\lambda_{k}^{0i}=\alpha^{i}\cdot G_{k}$, the value of $\alpha^{i}$ is determined by the system prior information. The formula of $G_{k}$ is as follows:

(47) $$\begin{array}{c} G_{k}=\frac{trace\left(N_{k}\right)}{\sum_{i=1}^{n}\alpha^{i}\cdot M_{k}^{ii}}, \end{array}$$

where $M_{k}^{ii}$ is the i-th element of the main diagonal of $M_{k}$, the calculation formulas of $N_{k}$ and $M_{k}$ are as follows:

(48) $$\begin{array}{c} N_{k}=B_{k}-H_{k-1}{\hat{Q}}_{k-1}H_{k-1}^{T}-\tau {\hat{R}}_{k}, \end{array}$$

(49) $$\begin{array}{c} M_{k}=\Phi_{k-1}P_{k:k}\Phi_{k-1}{}^{T}H_{k-1}^{T}H_{k-1}. \end{array}$$

In Equation (48), τ is the weakening factor and $B_{k}$ is unknown and can be estimated by the following formula:

(50) $$\begin{array}{c} B_{k}=\{\begin{array}{rr} \gamma_{k}\gamma_{k}^{T}, & k=1\\ \frac{\mu B_{k-1}+\gamma_{k}\gamma_{k}^{T}}{1+\mu }, & k>1 \end{array} \end{array}$$

where μ∈(0,1] is the forgetting factor.

Since $L_{md}$ is used to correct the one-step predicted error covariance matrix (PECM) in the prediction step of the filtering algorithm, the initial value ${\hat{R}}_{1}$ of the MNCM ${\hat{R}}_{k}$ must be set in advance, assuming that ${\hat{R}}_{1}$ also obeys the inverse Wishart distribution. The estimated value of measurement noise covariance matrix ${\hat{R}}_{k}$ in the time-update step is defined as:

(51) $$\begin{array}{c} {\hat{R}}_{k}=\{\begin{array}{rr} \frac{{\hat{T}}_{0}}{\left({\hat{t}}_{0}-m-1\right)}, & k=1\\ {\hat{R}}_{k-1}, & k>1 \end{array}, \end{array}$$

In Equation (51), the variation range of the slowly time-varying measurement noise covariance matrix is small, and the estimated value ${\hat{R}}_{k-1}$ at the previous time still has a great reference value for the current time estimation. Therefore, ${\hat{R}}_{k-1}$ estimated by the variational update recursively of the previous time is used as ${\hat{R}}_{k}$ at time k>1. ${\hat{R}}_{k}$ is used as the time-varying parameter of $L_{md}$ to modify the one-step predicted error covariance matrix (PECM) $P_{k:k-1}^{*}$ more accurately, and the accurate $P_{k:k-1}^{*}$ can affect the accuracy of the estimation result of the variational iteration recursion directly.

The multi-fading factor and updated monitoring strategy AKF-based variational Bayesian in this paper is composed of Equations (6), (12)–(14), (22)–(25), (27), (29)–(34), (41)–(51). The pseudo-code implementation of the proposed MFMS-VBAKF algorithm is listed in Algorithm 1.
**Algorithm 1** One time-step of the proposed multi-fading factor and updated monitoring strategy AKF-based variational Bayesian**Inputs:**$\;{\hat{X}}_{k-1:k-1}$,$\;P_{k-1:k-1}$,$\;{\hat{t}}_{k-1:k-1}$,$\;{\hat{T}}_{k-1:k-1}$, Φ, H,$\;Z_{k}$, m, n, ρ, b, μ, τ,$\;\alpha^{i}$, N**Time update**:**1.**$\;{\hat{X}}_{k:k-1}=\Phi X_{k-1:k-1}$,$\;\gamma_{k}=Z_{k}-{\hat{X}}_{k:k-1}$**2. if**k=1**then****3.** ${\hat{R}}_{k}={\hat{T}}_{0}/\left({\hat{t}}_{0}-m-1\right)$,$\;Q_{k}={\tilde{Q}}_{0}$**4.** $B_{k}=\gamma_{k}\gamma_{k}^{T}$,**5. else****6.** ${\hat{R}}_{k}={\hat{R}}_{k-1},\;Q_{k}={\hat{Q}}_{k-1}$**7.** $B_{k}=\mu B_{k-1}+\gamma_{k}\gamma_{k}^{T}/1+\mu$**8.**$\;N_{k}=B_{k}-H_{k-1}{\hat{Q}}_{k-1}H_{k-1}^{T}-\tau {\hat{R}}_{k}$,
**9.**$\;M_{k}=\Phi_{k-1}P_{k:k}\Phi_{k-1}{}^{T}H_{k-1}^{T}H_{k-1}$,**10.**$\;G_{k}=trace\left(N_{k}\right)/\overset{n}{\underset{i=1}{\sum }}\left(\alpha^{i}\cdot M_{k}^{ii}\right)$,$\;\lambda_{k}^{0i}=\alpha^{i}\cdot G_{k}$**11. if**$\lambda_{k}^{0i}>1$**12.**
 
$\lambda_{k}^{i}=\lambda_{k}^{0i}$
**13. else****14.**
 
$\lambda_{k}^{i}=1$
**15.**$\;L_{md}=diag\left[\lambda_{k}^{1},\lambda_{k}^{2},\cdots ,\lambda_{k}^{n}\right]$**16.**$\;d_{k}=\left(1-b\right)/\left(1-b^{k+1}\right)$**17.**$\;{\hat{Q}}_{k}=\left(1-d_{k}\right){\hat{Q}}_{k-1}+d_{k}\left(K_{k}\gamma_{k}\gamma_{k}^{T}K_{k}{}^{T}+P_{k}-\Phi_{k-1}P_{k-1}\Phi_{k-1}^{T}\right)$, c=1**18.**$\;\theta =min\left(eig\left(\;{\hat{Q}}_{k}\right)\right)$,**19.**$\;a_{k}=trace\left(P_{k}-\Phi_{k-1}P_{k-1}\Phi_{k-1}^{T}-{\hat{Q}}_{k-1}\right)/trace\left(K_{k}\gamma_{k}\gamma_{k}^{T}K_{k}{}^{T}\right)$**20.**$\;\beta_{k}=exp^{-a_{k}}$**21. while**θ<0**do****22.** ${\hat{Q}}_{k}={\hat{Q}}_{k-1}+d_{k}\left(K_{k}\gamma_{k}\gamma_{k}^{T}K_{k}{}^{T}+{\left(\beta_{k}\right)}^{p}\left(P_{k}-\Phi_{k-1}P_{k-1}\Phi_{k-1}^{T}-{\hat{Q}}_{k-1}\right)\right)$**23.** $\theta =min\left(eig\left(\;{\hat{Q}}_{k}\right)\right)$, c=c+1**24.**$\;P_{k:k-1}^{*}=L_{md}\Phi_{k-1}P_{k-1:k-1}\Phi_{k-1}{}^{T}+{\hat{Q}}_{k-1}$**Iterated measurement update****25. Initialization****26.**$\;V_{k}^{\left(i\right)}=\left(Z_{k}-H_{k}{\hat{X}}_{k}^{i}\right){\left(Z_{k}-H_{k}{\hat{X}}_{k}^{i}\right)}^{T}+H_{k}P_{k}H_{k}^{T}$**27.**$\;{\hat{t}}_{k}^{\left(i+1\right)}={\hat{t}}_{k:k-1}+1$, ${\hat{T}}_{k}^{\left(i+1\right)}=V_{k}^{\left(i\right)}+{\hat{T}}_{k}^{\left(i\right)}$**for **i=0:N−1**Update**$q^{\left(N\right)}\left(X_{k}\right)=\left(X_{k};{\hat{X}}_{k:k},P_{k:k}\right)$**given**$P_{k:k-1}^{*}$and$q^{\left(i+1\right)}\left(R_{k}\right)$:**28.**$\;E^{\left(i+1\right)}{\left[R_{k}^{-1}\right]}^{-1}={\left({\hat{t}}_{k}^{\left(i+1\right)}-m-1\right)}^{-1}{\hat{T}}_{k}^{\left(i+1\right)}$**29.**$\;{\hat{R}}_{k}^{\left(i+1\right)}=E^{\left(i+1\right)}{\left[R_{k}^{-1}\right]}^{-1}$**30.**$\;K_{k}^{\left(i+1\right)}=\;P_{k:k-1}^{*}H_{k}^{T}{\left(H_{k}P_{k:k-1}^{*}H_{k}^{T}+{\hat{R}}_{k}^{\left(i+1\right)}\right)}^{-1}$**31.**$\;{\hat{X}}_{k}^{\left(i+1\right)}={\hat{X}}_{k:k-1}+K_{k}^{\left(i+1\right)}\left(Z_{k}-H_{k}{\hat{X}}_{k:k-1}\right)$**32.**$\;{\hat{P}}_{k}^{\left(i+1\right)}=\left(I-K_{k}^{\left(i+1\right)}H_{k}\right)P_{k:k-1}{\left(I-K_{k}^{\left(i+1\right)}H_{k}\right)}^{T}+K_{k}^{\left(i+1\right)}{\hat{R}}_{k}^{\left(i+1\right)}{\left(K_{k}^{\left(i+1\right)}\right)}^{T}$**End for****33.**$\;{\hat{X}}_{k:k}=\;{\hat{X}}_{k:k}^{\left(N\right)}$, ${\hat{P}}_{k:k}=\;{\hat{P}}_{k:k}^{\left(N\right)}$, ${\hat{t}}_{k:k}=\;{\hat{t}}_{k}^{\left(N\right)}$, ${\hat{T}}_{k:k}={\hat{T}}_{k}^{\left(N\right)}$,$\;{\hat{R}}_{k}={\hat{R}}_{k}^{\left(N\right)}$**Outputs:**$\;{\hat{X}}_{k:k}$,$\;{\hat{P}}_{k:k}$,$\;{\hat{t}}_{k:k}$,$\;{\hat{T}}_{k:k}$,$\;{\hat{R}}_{k}$, ${\hat{Q}}_{k}$

## 4. Simulations and Results

The application of the proposed algorithm in target tracking is simulated. The target moves according to the continuous white noise accelerated motion model in the two-dimensional Cartesian coordinate system. Sensors are used to collect the target location. The system state is defined as $x_{k}=\left[x_{k}^{\prime }\;{\dot{x}}_{k}^{\prime }\;y_{k}^{\prime }\;{\dot{y}}_{k}^{\prime }\right]$, where $x_{k}^{\prime }\;$and$\;y_{k}^{\prime }$ represent the Cartesian coordinates of the target at time k,$\;{\dot{x}}_{k}^{\prime }\;$and ${\dot{y}}_{k}^{\prime }\;$represent the velocity of the target at the corresponding position [24,25]. The state transition matrix $\Phi_{k-1}$ and the measurement matrix $H_{k}$ are respectively set as:

(52) $$\begin{array}{c} \Phi_{k-1}=\left[\begin{array}{cc} I_{2} & \Delta tI_{2}\\ 0 & I_{2} \end{array}\right],\;H_{k}=\left[\begin{array}{cc} I_{2} & 0 \end{array}\right], \end{array}$$

where the parameter Δt=1 s represent the sampling interval, and $I_{n}$ represent the n-dimensional unit matrix. Similar to [25], the true process noise covariance matrix (PNCM) and the measurement noise covariance matrix (MNCM) are set to slow time-varying models, which are:

(53) $$\begin{array}{c} \{\begin{array}{c} \begin{array}{c} Q_{k}=\left(9.5+0.5cos\frac{\pi k}{T}\right)q\left[\begin{array}{cc} \frac{\Delta t^{3}}{3}I_{2} & \frac{\Delta t^{2}}{2}I_{2}\\ \frac{\Delta t^{2}}{2}I_{2} & \Delta tI_{2} \end{array}\right]\\ R_{k}=\left(0.1+0.05cos\frac{\pi k}{T}\right)r\left[\begin{array}{cc} 1 & 0.5\\ 0.5 & 1 \end{array}\right] \end{array} \end{array} \end{array}$$

The simulation environment is set as follows: T=1000 s is the total simulation time, q is a parameter related to process noise, and r is a parameter related to measurement noise. The fixed PNCM and MNCM are set as ${\tilde{Q}}_{k}=\sigma I_{4}$ and ${\tilde{R}}_{k}=\varepsilon I_{2}$, respectively, where σ and ε are the prior confidence parameters used to adjust the initial fixed noise covariance matrix.

The parameters in the MFMS-VBAKF algorithm proposed in this paper are set as follows: σ=1, ε=100, changing factor ρ=exp(−4), the number of variational iterations N=10, the initial value of the variational parameter ${\hat{t}}_{0}=1$,$\;{\hat{T}}_{0}=300I_{2}$, forgetting factor μ=0.95, the weakening factor τ=0.4, the parameter $\left[\alpha^{1}\;\alpha^{2}\;\alpha^{3}\;\alpha^{4}\right]=\left[1.7\;1.1\;1.7\;1.1\right]$, and the attenuation factor b=0.96.

This paper compares MFMS-VBAKF and true noise covariance matrix Kalman filter (TCMKF) [1], fixed noise covariance matrix Kalman filter (FCMKF) [1], SH-KF [15], ML-KF [20], ST-KF [21], and R-VBKF [26] algorithms. Table 1 lists the estimated parameters and parameter settings of the existing algorithms. All algorithms are programmed using MATLAB R2018a, and the simulation program runs on a computer with Intel^®^ Core™ i5-6300HQ CPU at 2.30 GHz and 8GB of RAM.

With the aim of evaluating the accuracy of system state estimation, the root mean square error and average root mean square error of position and velocity are regarded as performance indicators, which are defined as follows:

(54) $$\begin{array}{c} E_{RMSE,pos}≜\sqrt{\frac{1}{M}\overset{M}{\underset{S=1}{\sum }}\left({\left(x_{k}^{s}-{\hat{x}}_{k}^{s}\right)}^{2}+{\left(y_{k}^{s}-{\hat{y}}_{k}^{s}\right)}^{2}\right)} \end{array}$$

(55) $$\begin{array}{c} E_{ARMSE,pos}≜\sqrt{\frac{1}{MT}\overset{T}{\underset{k=1}{\sum }}\overset{M}{\underset{S=1}{\sum }}\left({\left(x_{k}^{s}-{\hat{x}}_{k}^{s}\right)}^{2}+{\left(y_{k}^{s}-{\hat{y}}_{k}^{s}\right)}^{2}\right)} \end{array}$$

where $\left(x_{k}^{’},{\hat{x}}_{k}^{’}\right)$ and $\left(y_{k}^{’},{\hat{y}}_{k}^{’}\right),$ respectively, represent the true value and estimated value of the position in the s-th Monte Carlo experiment and M=1000 represents the total number of Monte Carlo experiment runs. Similarly, the calculation formulas of RMSE and ARMSE for the corresponding velocity can be obtained.

**Figure 2:**
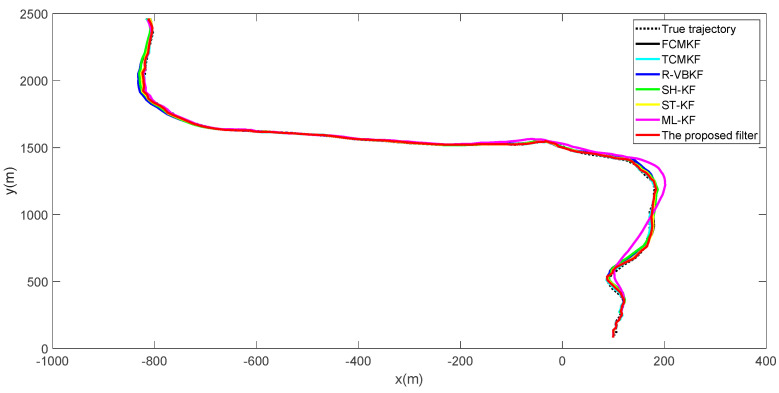
True and estimated trajectories of the target.

True and estimated trajectories of the target are shown in Figure 2. It can be seen that, compared to the existing adaptive filtering algorithm, the proposed filter maintains excellent target tracking performance throughout the entire process.

**Figure 3:**
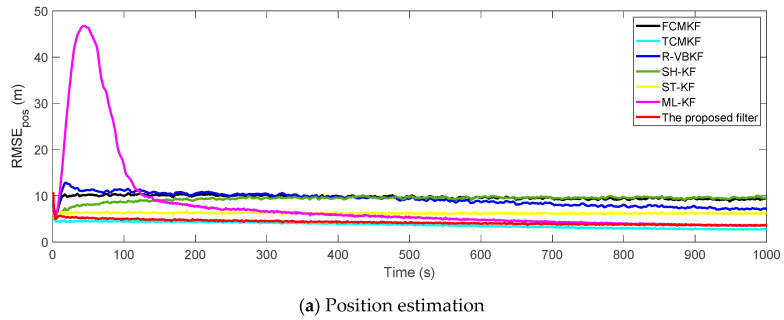

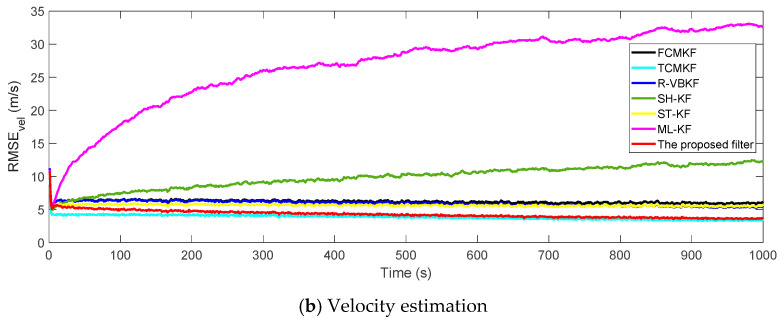
The root mean square errors (RMSEs) of the target position and velocity estimation.

Figure 3a,b plot the RMSE variation curves of the position and velocity of the existing filter and the proposed MFMS-VBAKF, respectively. The RMSE of the estimation result of TCMKF algorithm is regarded as the benchmark. It can be seen from Figure 2 that compared with existing algorithms, the proposed algorithm has a faster convergence speed and higher accuracy. To further elaborate on the advantages of the proposed algorithm, Table 2 lists the average root mean square error (ARMSE) of different KF filtering algorithms:

According to the data in Table 2, it can be found that in comparison with other AKF algorithms, the MFMS-VBAKF algorithm has the smallest ARMSE and the highest accuracy in estimating target position and velocity.

To compare the computational complexity with existing algorithms, Table 3 lists the single-step running time of each algorithm. It can be seen that, compared with the R-VBAKF using the variational Bayesian method, the proposed MFMS-VBAKF increases the single-step running time by 0.21 μs. The design of multi-fading factors and updated monitoring strategy has ensured a substantial increase in estimation accuracy while bringing higher computational complexity.

**Figure 4:**
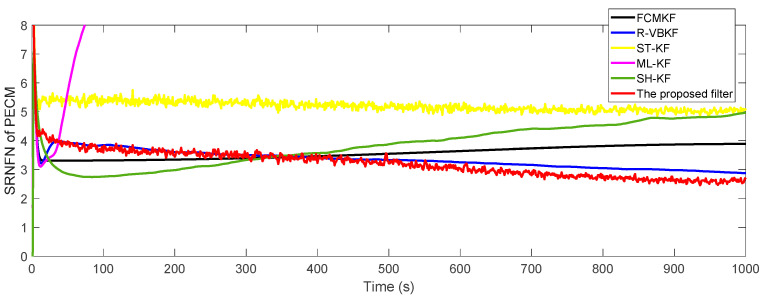
The SRNFN of PECM estimation.

To evaluate the accuracy of the estimation of one-step predicted state error covariance matrix PECM and the noise covariance matrices PNCM, MNCM, the square root of the normalized Frobenius norm (SRNFN) and the averaged SRNFN (ASRNFN) are used as the measure of error, which are defined as:

(56) $$\begin{array}{c} E_{SRNFN,P}≜{\left(\frac{1}{n^{2}M}\overset{M}{\underset{s=1}{\sum }}||{\hat{P}}_{k:k-1}^{s}-{\hat{P}}_{o,k:k-1}^{s}||{}^{2}\right)}^{1/4} \end{array}$$

(57) $$\begin{array}{c} E_{ASRNFN,P}≜{\left(\frac{1}{n^{2}MT}\overset{T}{\underset{k=1}{\sum }}\overset{M}{\underset{s=1}{\sum }}||{\hat{P}}_{k:k-1}^{s}-{\hat{P}}_{o,k:k-1}^{s}||{}^{2}\right)}^{1/4} \end{array}$$

where ${\hat{P}}_{o,k:k-1}^{s}$ and ${\hat{P}}_{k:k-1}^{s}$ represent the true value and estimated value of the noise covariance matrix or one-step predicted state error covariance matrix in the s-th Monte Carlo experiment, respectively. The SRNFN and ASRNFN of the estimation result of PECM are shown in Figure 4 and Table 4, respectively.

It can be clearly seen that, compared with the existing adaptive KF algorithm, if the noise covariance matrices are slowly time-varying, the SRNFN of the MFMS-VBAKF algorithm is smaller than the SRNFN of the current algorithm. Compared with R-VBKF with similar performance, the ASRNFN of MFMS-VBAKF is reduced by 5.45%.

**Figure 5:**
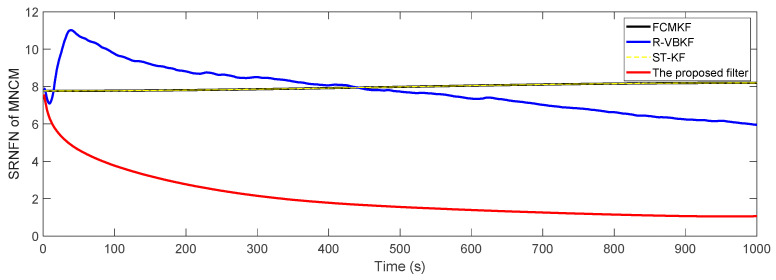
The square root of the normalized Frobenius norm (SRNFN) of the measurement noise covariance matrix (MNCM) estimation.

Figure 5 shows the SRNFN of the measurement noise covariance matrix (MNCM) estimation. Obviously, the MFMS-VBAKF algorithm has the strongest tracking ability, the highest estimation accuracy and the fastest convergence speed of the slowly time-varying measurement noise covariance matrix estimation.

**Figure 6:**
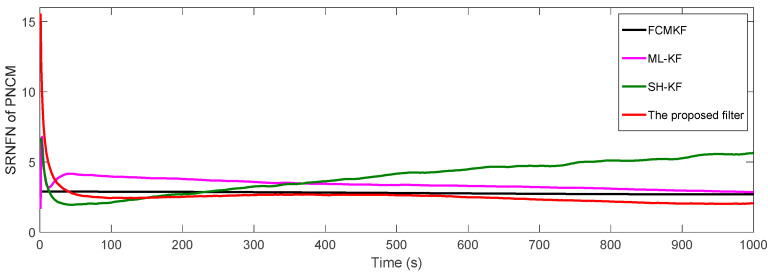
The SRNFN of the PNCM estimation.

Figure 6 and Table 5 show respectively the SRNFNs and the ASRNFNs of the PNCM from the existing filters and MFMS-VBAKF algorithm. It can be seen that the SRNFN and ASRNFN of the proposed MFMS-VBAKF are both smaller than the current filters. Thus, the MFMS-VBAKF has better estimation accuracy and satisfactory convergence speed in PNCM estimation.

Next, we compare and analyze the influence of the values of four critical parameters (changing factor ρ, forgetting factor μ, weakening factor τ and attenuation factor b) in the MFMS-VBAKF algorithm on the estimated effect.

**Figure 7:**
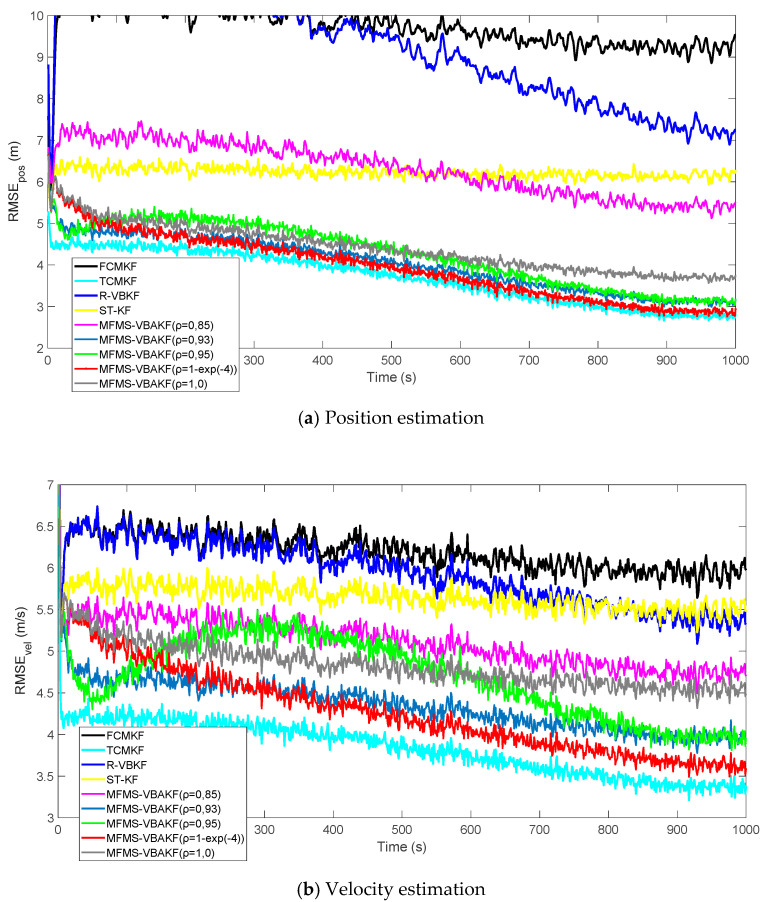
The RMSEs of the position and velocity estimation in the case of ρ=0.85, 0.93, 0.95, 1−exp(−4),1.0.

Figure 7 shows the RMSEs of position and velocity from the existing filters and MFMS-VBAKF in the case of ρ=0.85, 0.93, 0.95, 1−exp(−4),1.0. The MFMS-VBAKF with ρ=0.85, 0.93, 0.95, 1−exp(−4), 1.0 has better estimation accuracy than existing adaptive KF filters. And when ρ=1−exp(−4), the MFMS-VBAKF algorithm has the best estimation accuracy and convergence performance.

**Figure 8:**
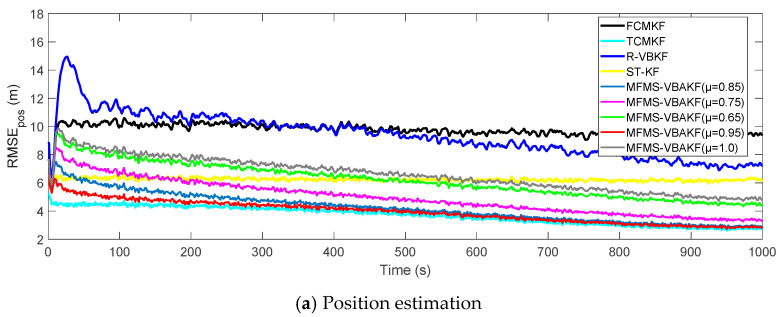

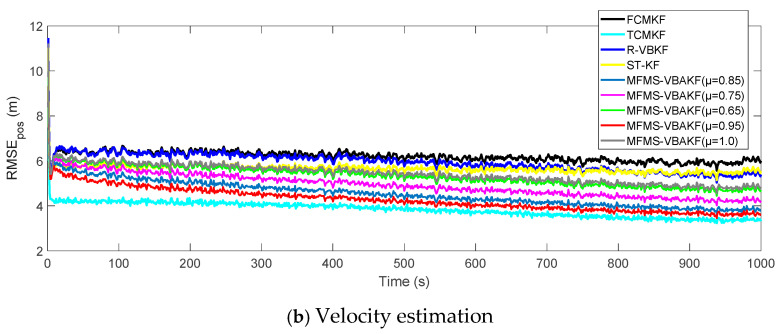
The RMSE of the position and velocity estimation in the case of μ = 0.65, 0.75, 0.85, 0.95, 1.0.

Figure 8 plots the RMSE curves of position and velocity from the existing filters and the MFMS-VBAKF in the case of μ=0.65, 0.75, 0.85, 0.95, 1.0.The MFMS-VBAKF with μ=0.65, 0.75, 0.85, 0.95, 1.0. has better estimation accuracy than existing adaptive KF filters, and when μ=0.95, the MFMS-VBAKF algorithm has the best estimation accuracy and convergence performance.

**Figure 9:**
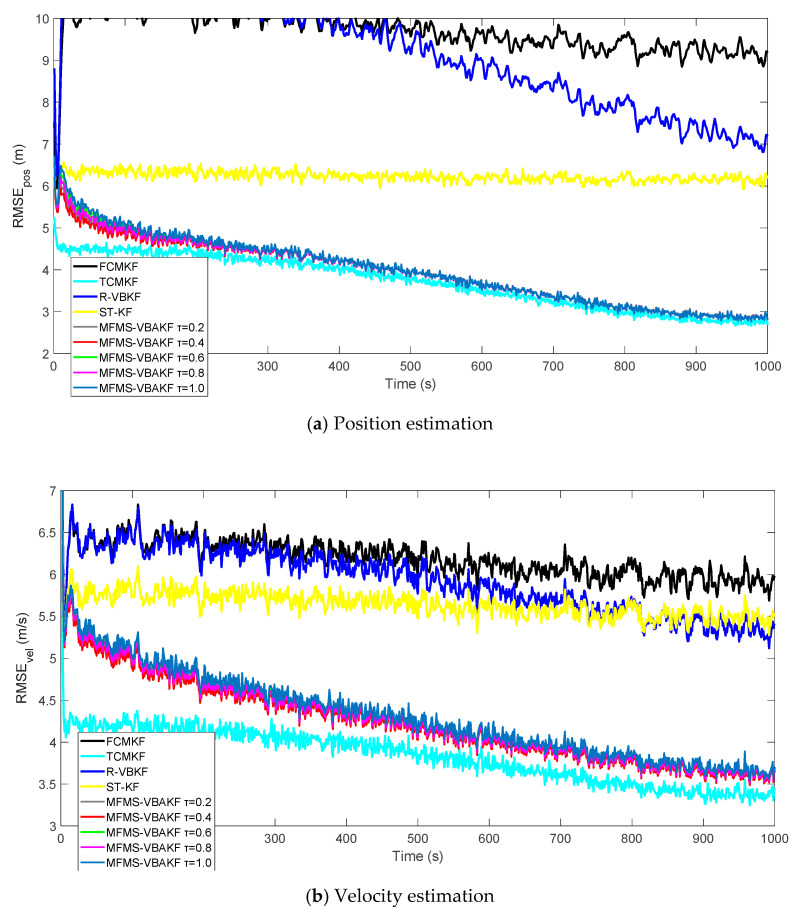
The RMSE of the position and velocity estimation in the case of τ=0.2, 0.4, 0.6, 0.8, 1.0.

Figure 9 plots the RMSE curves of position and velocity from the existing filters and the MFMS-VBAKF in the case of τ=0.2, 0.4, 0.6, 0.8, 1.0. The MFMS-VBAKF with τ=0.2, 0.4, 0.6, 0.8, 1.0 has better estimation accuracy than existing adaptive KF filters, and when τ= 0.4, the MFMS-VBAKF algorithm has the best estimation accuracy and convergence performance.

**Figure 10:**
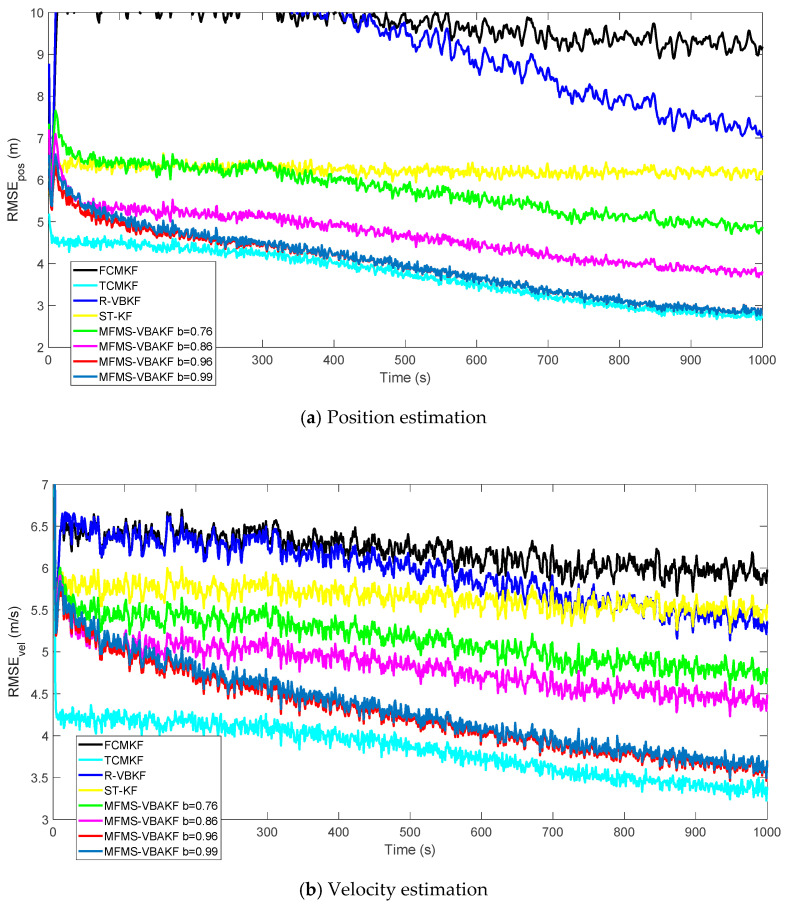
The RMSE of the position and velocity estimation in the case of b=0.76, 0.86, 0.96, 0.99.

Figure 10 shows the RMSEs of position and velocity from the existing filters and MFMS-VBAKF in the case of b=0.76, 0.86, 0.96, 0.99. The MFMS-VBAKF with b=0.76, 0.86, 0.96, 0.99. has better estimation accuracy than existing adaptive KF filters. And when b=0.96, MFMS-VBAKF algorithm has the best estimation accuracy and convergence performance.

For the sake of testing the robustness of the adaptive correction capability of the MFMS-VBAKF algorithm when the fixed noise covariance matrices are set to different initial values, the priori confidence parameters σ and ε are set to change in combination within the grid area of (σ,ε)∈[0.1, 800]×[0.1, 800]. The ARMSEs estimated by the algorithm for position and velocity are displayed in Figure 10.

**Figure 11:**
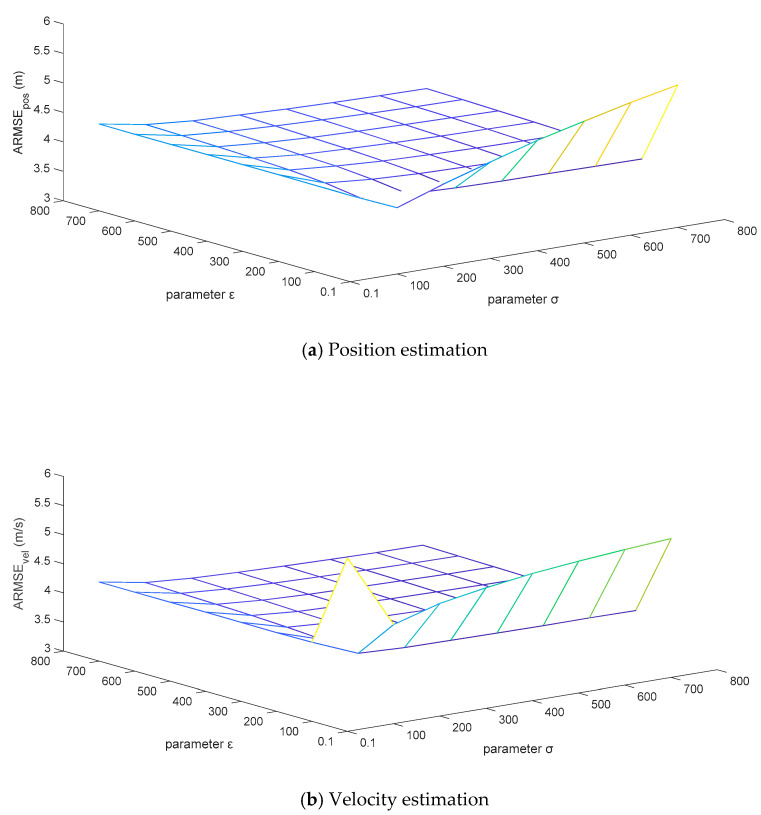
The ARMSEs of the position and velocity estimation under a combination of (σ,ε)∈[0.1, 800]×[0.1, 800].

It can be analyzed from Figure 11 that the ARMSEs of position and velocity estimation are flat in a large area of the set grid, and the estimation results are close to the actual values. However, the initial setting values of the fixed noise covariance matrices in the extremely narrow area on the right edge of the grid are too different from the actual values, which leads to unsatisfactory performance of the estimation results. This is caused by the variational Bayesian method that can only guarantee local convergence. In general, the estimated effects of the MFMS-VBAKF algorithm can converge to near the actual values, with excellent robust performance.

## 5. Conclusions

This paper presents a multi-fading factor and updated monitoring strategy AKF-based variational Bayesian with the inaccurate time-varying process and measurement noise covariance matrices. The model of measurement error is defined as the inverse Wishart distribution, and the variational Bayesian method is used to recursively estimate the measurement noise covariance matrix and the system state, the estimation results of the two can be approximated to the true value. The process noise covariance matrix is estimated by the maximum a posteriori principle, and the updated monitoring strategy with adjustment factors is used to guarantee the positive semi-definiteness of the updated matrix. The estimated value of measurement noise covariance obtained by the variational iteration recursion and the estimated value of process noise covariance obtained by updating the monitoring strategy are used as time-varying parameters of multiple fading factors, which can be corrected to obtain more accurate state predicted error covariance. Variational Bayesian and the updated monitoring strategy and multi-fading factors complement each other, which not only enhances the responsiveness of target tracking, but also improves the estimation accuracy of variational iteration recursion. The simulation results show that the proposed MFMS-VBAKF algorithm realizes the simultaneous estimation of the process noise covariance matrix and the measurement noise covariance matrix, and has achieved satisfactory results in terms of estimation accuracy, convergence performance, and robustness.

## 6. Future Work

Compared with existing filtering algorithms, the MFMS-VBAKF algorithm proposed in this paper exhibits excellent performance in the state estimation problem of linear systems with inaccurate time-varying processes and measurement noise covariance matrix. However, in real applications, the influence of the control input of the system should not be underestimated. Control input will cause large changes in noise, and even noise will obey non-Gaussian distribution. Moreover, most of the systems in real applications are nonlinear systems. In the above cases, MFMS-VBAKF will not be applicable. Therefore, in future work, the theory of this paper will be further extended to the problem of nonlinear adaptive Kalman filtering. The problem of nonlinear AKF with inaccurate state transition matrix and non-Gaussian noise distribution will be further studied.

## Figures and Tables

**Table 1:** Estimated parameters and parameter settings of the existing algorithms.

Types of Filtering Algorithms	Estimated Parameters	Algorithm Parameters	Value of Algorithm Parameters
SH-KF	${\hat{X}}_{k},\;{\hat{P}}_{k:k-1}$,${\hat{P}}_{k},\;{\hat{Q}}_{k}$	Forgetting factor	0.96
ML-KF	${\hat{X}}_{k},\;{\hat{P}}_{k:k-1}$,$\;{\hat{P}}_{k},{\hat{Q}}_{k}$	The size of sliding window	150
ST-KF	${\hat{X}}_{k},\;{\hat{P}}_{k:k-1}$,$\;{\hat{P}}_{k}$	Forgetting factor	0.94
R-VBKF	${\hat{X}}_{k},\;{\hat{P}}_{k:k-1}$,${\hat{P}}_{k},\;{\hat{R}}_{k}$	The number of iterations	10
		Forgetting factor	0.98

SHKF, Sage–Husa Kalman filter in [15]; MLKF, maximum likelihood Kalman filter in [20]; ST-KF, strong-tracking Kalman Filter in [21]; R-VBKF, the Kalman filter algorithm that uses variational iteration to recursively estimate ${\hat{R}}_{k}$ and ${\hat{X}}_{k}$ in [26].

**Table 2:** Average root mean square error (ARMSE) of various Kalman filter algorithms.

Filters	$E_{ARMSE,pos}/m$	$E_{ARMSE,vel}/\left(m\cdot s^{-1}\right)$
FCMKF	9.853	6.348
ML-KF	4.596	29.759
SH-KF	9.646	9.545
R-VBKF	9.352	5.961
ST-KF	6.157	5.653
MFMS-VBAKF	4.073	3.946
TCMKF	3.649	3.253

**Table 3:** Single-step running time of each algorithm.

Filters	Single-Step Running Time (μs)
FCMKF	0.28
ST-KF	0.41
SH-KF	0.46
ML-KF	0.54
R-VBKF	0.71
MFMS-VBAKF	0.92

**Table 4:** ASRNFN of PECM estimation of various Kalman filter algorithms.

Filters	$E_{ASRNFN,P}$
ML-KF	21.365
ST-KF	5.573
SH-KF	4.113
FCMKF	3.852
R-VBKF	3.115
MFMS-VBAKF	2.945

**Table 5:** The averaged SRNFN (ASRNFN) of PNCM estimation of various Kalman filter algorithms.

Filters	$E_{ASRNFN,Q}\;$
SH-KF	4.915
ML-KF	3.688
FCMKF	3.357
MFMS-VBAKF	2.754

## Data Availability

No new data were created or analyzed in this study. Data sharing is not applicable to this article.

## Appendix A. Derivation of (21)

The Bayesian estimation theory states that the joint PDF can be defined as:

(A1) $$\begin{array}{c} p\left(X_{k},R_{k},\;Z_{1:k}\right)=p\left(Z_{k}|X_{k},R_{k}\right)p\left(X_{k}|Z_{1:k-1}\right)p\left(R_{k}|Z_{1:k-1}\right)p\left(Z_{1:k-1}\right) \end{array}$$

Equations (3), (4), and (11) are substituted into (A1) to derive:

(A2) $$\begin{array}{c} p\left(X_{k},R_{k},\;Z_{1:k}\right)=N\left(Z_{k};H_{k}X_{k},R_{k}\right)N\left(X_{k-1};{\hat{X}}_{k:k-1},P_{k:k-1}\right)IW\left(R_{k-1};{\hat{t}}_{k-1:k-1},{\hat{T}}_{k-1:k-1}\right)p\left(Z_{1:k-1}\right) \end{array}$$

Combined with Equation (5), logN(G;μ,Σ) can be rewritten as:

(A3) $$\begin{array}{c} \log N\left(G;\mu ,\Sigma \right)=\log\left[\frac{1}{\sqrt{\left|2\pi \Sigma \right|}}exp^{-\frac{1}{2}{\left(G-\mu \right)}^{Τ}\Sigma^{-1}\left(G-\mu \right)}\right]\\ =\log\frac{1}{\sqrt{2\pi }}-\frac{1}{2}\log\left|\Sigma \right|-\frac{1}{2}{\left(G-\mu \right)}^{T}\Sigma^{-1}\left(G-\mu \right)\\ =-\frac{1}{2}\log\left|\Sigma \right|-\frac{1}{2}{\left(G-\mu \right)}^{T}\Sigma^{-1}\left(G-\mu \right)+c_{G}, \end{array}$$

where $c_{G}$ denotes a constant related to the variable G.

Combined with Equation (8), logIW(C;λ,Ψ)can be rewritten as:

(A4) $$\begin{array}{c} \log IW\left(C;\lambda ,\Psi \right)=\log\left\{\frac{{\left|\Psi \right|}^{\lambda /2}}{2^{\lambda d/2}\Gamma_{d}\left(\frac{\lambda }{2}\right)}{\left|C\right|}^{-\left(\lambda +d+1\right)/2}exp^{-\frac{1}{2}tr\left[\Psi C^{-1}\right]}\right\}\\ =\frac{\lambda }{2}\log\left|\Psi \right|-\frac{\left(\lambda +d+1\right)}{2}\log\left|C\right|-\frac{1}{2}tr\left[\Psi C^{-1}\right]-\frac{\lambda d}{2}\log2-\log\left|\Gamma_{d}\left(\frac{\lambda }{2}\right)\right|\\ \begin{array}{c} =-\frac{\left(\lambda +d+1\right)}{2}\log\left|C\right|-\frac{1}{2}tr\left[\Psi C^{-1}\right]+c_{C}, \end{array} \end{array}$$

where $c_{C}$ denotes the constant related to the variable C.

Combining (A2)–(A4), the logarithmic form of the joint PDF can be written as:

(A5) $$\begin{array}{c} \log p\left(X_{k},R_{k},\;Z_{1:k}\right)=\log\{N\left(Z_{k};H_{k}X_{k},R_{k}\right)N\left(X_{k-1};{\hat{X}}_{k:k-1},P_{k:k-1}\right)\\ \times IW\left(R_{k-1};{\hat{t}}_{k-1:k-1},{\hat{T}}_{k-1:k-1}\right)p\left(Z_{1:k-1}\right)\\ =-\frac{1}{2}\log\left|R_{k}\right|-\frac{1}{2}{\left(Z_{k}-H_{k}X_{k}\right)}^{T}R_{k}^{-1}\left(Z_{k}-H_{k}X_{k}\right)-\frac{1}{2}\log\left|P_{k:k-1}\right|\\ -\frac{1}{2}{\left(X_{k}-{\hat{X}}_{k:k-1}\right)}^{T}P_{k:k-1}^{-1}\left(X_{k}-{\hat{X}}_{k:k-1}\right)-\frac{\left({\hat{t}}_{k:k-1}+m+1\right)}{2}\log\left|R_{k}\right|-\frac{1}{2}tr\left({\hat{T}}_{k:k-1}R_{k}^{-1}\right)+c_{\emptyset }\\ =-\frac{\left({\hat{t}}_{k:k-1}+m+2\right)}{2}\log\left|R_{k}\right|-\frac{1}{2}{\left(Z_{k}-H_{k}X_{k}\right)}^{T}R_{k}^{-1}\left(Z_{k}-H_{k}X_{k}\right)-\frac{1}{2}tr\left({\hat{T}}_{k:k-1}R_{k}^{-1}\right)\\ -\frac{1}{2}\log\left|P_{k:k-1}\right|-\frac{1}{2}{\left(X_{k}-{\hat{X}}_{k:k-1}\right)}^{T}P_{k:k-1}^{-1}\left(X_{k}-{\hat{X}}_{k:k-1}\right)+c_{\emptyset } \end{array}$$

where $c_{\emptyset }$denotes a constant related to the variables $X_{k}$ and $R_{k}$.

Bring (A5) to Equation (20) to get the further derivation of Equation (21):

(A6) $$\begin{array}{c} \log q^{\left(i+1\right)}\left(R_{k}\right)=E_{X_{k}}^{\left(i\right)}\left[\log p\left(X_{k},R_{k},\;Z_{1:k}\right)\right]+c_{R_{k}}\\ =-\frac{\left({\hat{t}}_{k:k-1}+m+2\right)}{2}\log\left|R_{k}\right|-\frac{1}{2}E^{\left(i\right)}\left[tr\left(\left(V_{k}^{\left(i\right)}+{\hat{T}}_{k:k-1}\right)R_{k}^{-1}\right)\right]-\frac{1}{2}E^{\left(i\right)}\left[tr\left({\hat{T}}_{k:k-1}R_{k}^{-1}\right)\right]\\ -\frac{1}{2}E^{\left(i\right)}\left[{\left(Z_{k}-H_{k}X_{k}\right)}^{T}R_{k}^{-1}\left(Z_{k}-H_{k}X_{k}\right)\right]-\frac{1}{2}E^{\left(i\right)}\left[{\left(X_{k}-{\hat{X}}_{k:k-1}\right)}^{T}P_{k:k-1}^{-1}\left(X_{k}-{\hat{X}}_{k:k-1}\right)\right]\\ -E^{\left(i\right)}\left[\frac{1}{2}\log\left|P_{k:k-1}\right|\right]+c_{\emptyset }\\ =-\frac{\left({\hat{t}}_{k:k-1}+m+2\right)}{2}\log\left|R_{k}\right|-\frac{1}{2}tr\left(\left(V_{k}^{\left(i\right)}+{\hat{T}}_{k:k-1}\right)R_{k}^{-1}\right)+c_{R_{k}} \end{array}$$

where

(A7) $$\begin{array}{c} c_{R_{k}}=-E^{\left(i\right)}\left[\frac{1}{2}\log\left|P_{k:k-1}\right|\right]-\frac{1}{2}E^{\left(i\right)}\left[{\left(Z_{k}-H_{k}X_{k}\right)}^{T}R_{k}^{-1}\left(Z_{k}-H_{k}X_{k}\right)\right]+c_{\emptyset }. \end{array}$$

Equation (26) can be derived in a similar way.
